# Round the Hospitals

**Published:** 1921-04-16

**Authors:** 


### ROUND THE HOSPITALS.

We are glad to learn that in preparation for the Conference on nurse-training called by the General Nursing Council for April 28, a meeting of the Poor-law Infirmary Matrons' Association will be held on Tuesday, April 26, at 3 p.m., at the Eustace Miles Restaurant, Chandos Street, Charing Cross, to discuss the draft syllabus and the best means of helping the smaller training-schools. All matrons and superintendent nurses of infirmaries recognised by the Ministry of Health will be welcomed. Such a preliminary discussion should prove of real service in clearing the ground and saving the time of the main Conference. We hope other bodies of nurses will follow this lead.

**ROUND THE HOSPITALS**—(*continued*).

The questionnaire circulated by the General Nursing Council for Scotland to general hospitals, fever hospitals, and mental hospitals respectively is very much on the same lines as that adopted by the General Nursing Council for England and Wales. It is concise and clear, and with few exceptions the questions set do not comprise more information than is readily available without long calculations. We are not sure that the ratio of sisters, staff nurses, and probationers to patients will be a welcome requirement, but there is no doubt it is a highly instructive bit of information on which to base comparisons. In the form for mental hospitals we should like to have seen a query relating to the number, if any, of general-trained nurses, and in particular whether the matron or head nurse has this qualification.

---

The official announcement of the establishment of "Queen Alexandra's Military Families' Nursing Service" marks another advance in the attitude of the War Office towards the wives and children of Service men. For some considerable time there has been a permanent establishment of the Military Families' and Military Isolation Hospitals, and these will now be taken over by the new service, which will consist of matrons, sisters-in-charge, and staff nurses. Appointments will be made under regulations approved by the Army Council. The rates of pay are to be the same as in Q.A.I.M.N.S., with an additional £20 a year for sisters-in-charge up to thirty beds and £30 a year over thirty beds. Outdoor uniform is being considered, and uniform allowance for the first year will be £20, for the second £5, and for succeeding years £10.

---

The prospectus has been issued of the United Nursing Services Club, Ltd., with a share capital of £30,000 divided into 15,000 shares of £2 each. This company has been formed, as already announced in these columns, to provide and equip a ladies' club for the use of past, present, and future members of the naval and military nursing and kindred services. Already 1,241 applications for membership have been received, and negotiations are approaching completion for the purchase of 34 Cavendish Square for the club premises. The purchase price (£12,000) will be paid for out of the grant of £15,000 from the United Services Fund.

---

It is proposed that the entrance fee for original members of the United Nursing Services Club shall be £1 1s., and that it shall be waived in the case of members who take more than one share in the company. The annual subscription will be £2 12s. 6d., £1 11s. 6d., and 10s. 6d. for town, country, and overseas members respectively, and half-a-guinea less in the case of those town and country members who have already applied for membership. The directors of the company are Dame Ethel Becher, Dame S. E. Oram, Miss McCall Anderson, Miss M. A. Gadsden, Miss S. M. Steer, and Miss B. M. Miller, only one of whom will receive remuneration for her services unless the company, in general meeting, votes such remuneration. The directors and their friends have applied for 3,629 shares, and over 10,000 shares remain for subscription before July 1, 1921. The net profits of the company will be applied first to paying a dividend of not more than 7 per cent. on the shares. The Secretary of the company is Miss Gertrude Richards, C.B.E., R.R.C., and the registered office is 34 Cavendish Square, W. 1.

---

Princess Christian has procured a magnificent gift for the Royal British Nurses' Association, of which her Royal Highness was foundress, and has remained always President. The lease of 194 Queen's Gate, formerly Queen Mary's Hostel for Nurses, has been taken over by the Association, and the Committee of the Hostel have generously handed over the whole of the furniture and equipment there to the Chartered Association of Nurses. The Royal British Nurses' Association has long felt the need of a club for its members, and could hardly have secured more commodious or charmingly situated premises. In making this auspicious announcement to the members Her Royal Highness desires it to be known that it has always been her particular wish that the club which it is proposed to establish as part of the work of the Association shall be available for all trained nurses, whether members of the Royal British Nurses' Association or not. Princess Christian also expresses the wish that Australian nurses—and, indeed nurses from all the Dominions—may always find a special welcome at the new club.

---

Further steps to consider the provision of nursing services for the insured out of the valuation surplus or under Section 21 of the 1911 Health Insurance Act have been taken. It will be remembered that the question was raised by Mr. David Pennant of the Queen Victoria Jubilee Institute Committee at the meeting of the Central Council for District Nursing on February 28. Meetings have since been held between representatives of the Q.V.J.I.N. and of the approved societies, and the Joint Committee of Approved Societies and the National Conferences of Industrial Societies, and a draft agreement for submission to all District Nursing Associations has been approved. It suggests a *per capita* basis of payment, that the charge to approved societies should be a flat rate based upon the average cost of nursing services in the area, possibly some figure between 1s. and 1s. 4d. per visit, and that the best authority for making the necessary arrangements for London would be the Central Council for District Nursing. We congratulate the District Nursing Authorities on the progress that has thus been made.

---

In the new Central Midwives Board, now being constituted, the Royal British Nurses' Association will have no direct representative. But one of the two representatives appointed by the Ministry of Health is Miss Gladys le Geyt, R.B.N.A., one of

**ROUND THE HOSPITALS**—(*continued*).

two nurse-midwives nominated by the Association at the request of Dr. Addison for him to select from. Miss le Geyt, who was trained at St. Bartholomew's Hospital, is superintendent of an infant-welfare centre. The second nominee of the Ministry of Health is Miss Olive Haydon, who is an approved teacher of midwifery and the superintendent of a training-school for midwives. The Society of the Medical Officers of Health have nominated Dr. Robert Lyster, Medical Officer of Health for Hampshire, as their representative.

Few hospital "grievances" awake more cordial sympathy in the public than the bad organisation which leads to waking patients very early in the morning to go through their ablutions. At an inquest recently held at Stepney on an inmate of the infirmary, aged seventy-four, one of the nurses, in giving evidence, stated that she first noticed the old lady was ill when she was engaged in washing the patients at 3.20 A.M. This practice has been reprobated again and again as cruel and needless by doctors and matrons alike. But short-handed night nurses drift back to it from sheer compulsion of time. A writer in the *Manchester Guardian* has described the process with a wealth of literary allusion, and concludes that "the victims, conferring afterwards with bated breath among themselves, like the huddled patients just emerged from a sheep-wash, have generally understood that their bane was a system which kept a night staff on until about seven or eight, and ordained that it must not go off duty till every one washable in a ward had been washed."

The Grahamstown Hospital Board has rescinded its resolution censuring Miss Gordon, matron of the Albany General Hospital, in connection with the sanitary and nursing arrangements of the hospital. This has caused universal satisfaction. Miss Gordon is popular with her staff, and the Government Commissioners of Inquiry paid a deserved tribute to their good discipline. We commented in our issue of March 19 on the findings of the Commissioners, and are glad that the Grahamstown Board have confessed the mistake they made in censuring one of their officials before the complaints had been properly investigated. Had they refused to rescind the resolution they would have lost the services of a very capable matron.

At a Conference held in Bombay between the Association of Nursing Superintendents of India and the Association of Trained Nurses of India it has been decided to amalgamate the two Societies with a view to enlarging their scope, and to appoint a paid secretary, who shall also be editor of the *Nursing Journal of India*, with headquarters in Delhi or, during the hot weather, in Simla. An appeal is to be made to each Provincial Government and to the Government of India for funds, for it is estimated that Rs.100,000 (one lakh) will be required to put the new Association on a sound financial basis. There is a strong desire among the members to obtain State Registration. The shortage of probationers is acute, and private nursing is gravely hampered for lack of well-organised nurses' co-operations in the towns. It is evident the joint Association has a wide field of work.

